# Sublingual Administration of All-Trans Retinoic Acid in Two Patients With Acute Promyelocytic Leukemia and Previous Gastric Bypass: A Case Series

**DOI:** 10.7759/cureus.109775

**Published:** 2026-05-27

**Authors:** Samuel Srinivasan, Rand Al-Adayleh, Zeinab Nasser, Vijayalakshmi Donthireddy

**Affiliations:** 1 Internal Medicine, Henry Ford Health System, Detroit, USA; 2 Hematology/Oncology, Henry Ford Health System, Detroit, USA

**Keywords:** acute myeloid leukemia (aml), acute promyelocytic leukemia (apl), all-trans retinoic acid, atra, malignant hematology

## Abstract

Standard treatments for acute promyelocytic leukemia (APL) are well established and highly effective, including all-trans retinoic acid (ATRA), which is an orally administered drug that targets the hallmark APL-associated PML:RARA fusion protein. However, patients who have undergone gastric bypass may have reduced oral drug bioavailability due to altered gastrointestinal anatomy, and sublingual rather than oral administration of ATRA may be required to optimize systemic absorption. Here, we describe two patients with APL who had previously undergone gastric bypass surgery and had a suboptimal initial response to oral ATRA therapy. Both patients showed substantial improvement in regard to their complete blood count parameters after switching to sublingual ATRA, suggesting that this route of administration may be needed for individuals with APL who have altered gastrointestinal anatomy that may lead to suboptimal bioavailability. Overall, the pharmacodynamics of ATRA in patients with conditions affecting systemic drug absorption require further investigation.

## Introduction

Acute promyelocytic leukemia (APL) is a subtype of acute myeloid leukemia (AML), accounting for approximately 10% to 15% of adult AML cases per year [1]. Historically, APL was considered the most fatal form of AML; however, therapeutic advances have greatly improved prognoses, particularly due to the cornerstone treatment all-trans retinoic acid (ATRA), which is typically administered orally alongside other agents [2,3]. However, certain patients may have limited systemic absorption of orally administered ATRA, including those with gastrointestinal disorders. Patients with a history of gastric bypass represent a growing population in whom there is concern regarding the ability to absorb various medications. Therefore, although a sublingual formulation is not commercially available, sublingual ATRA administration might optimize drug delivery in certain patients.

Here, we report two patients with APL and a history of gastric bypass surgery who were treated with sublingual ATRA after initial suboptimal responses (no improvement in cell counts) to oral administration. Both patients demonstrated an inadequate response to traditional oral therapy, responding only after the change was made to a sublingual formulation. Given the limited literature regarding patients with APL and similar constraints, these cases highlight important examples of the efficacy of sublingual ATRA in such patients.

## Case presentation

Case 1

A 36-year-old woman presented to the Emergency Department (ED) for a syncopal episode. The patient had a history of obesity and had undergone gastric bypass surgery 13 months earlier. Laboratory test results showed pancytopenia as documented in Table 1. Results from bone marrow biopsy (morphology: decreased trilineage hematopoiesis largely replaced by sheets of blasts with abundant pink cytoplasm, 90% promyelocytes) and flow cytometry (bone marrow origin, positive for increased myeloblasts) confirmed APL, as shown in Figure 1, Figure 2, and Figure 3 below. The patient was started on oral ATRA (40 mg twice daily), followed by arsenic trioxide the next day.

**Table 1:** Complete blood count Pancytopenia was present at presentation with diminished cell counts across all cell lines.

	Normal limits	Actual count
Platelets	150-450 K/uL	20 k/uL (low)
White blood cell count	3.8-10.6 K/uL	0.9 k/uL (low)
Absolute neutrophil count	1.8-7.7 K/uL	0.5 k/uL (low)
Hemoglobin	13.5-17.0 g/dL	9.3 g/dL (low)

**Figure 1:**
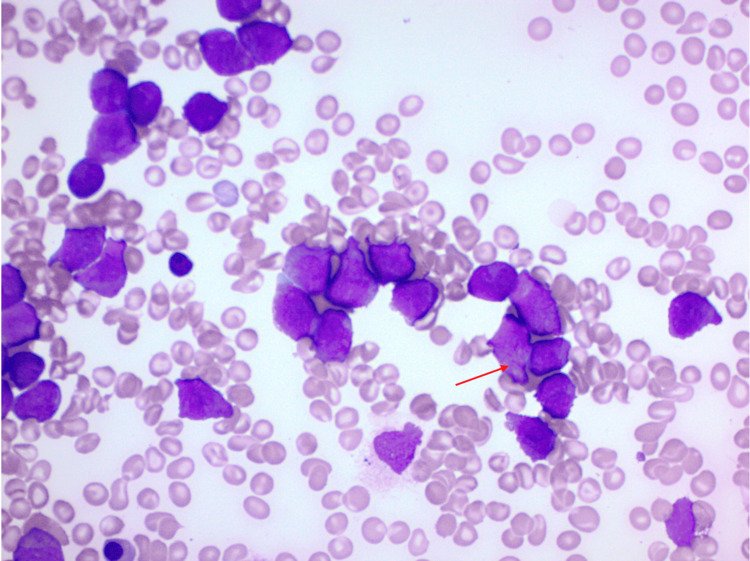
High-power examination of bone marrow aspirate smear Abnormal promyelocytes with kidney-shaped or bilobed nuclei, cytoplasmic pink granules, and Auer rods (red arrow) at 50× magnification.

**Figure 2:**
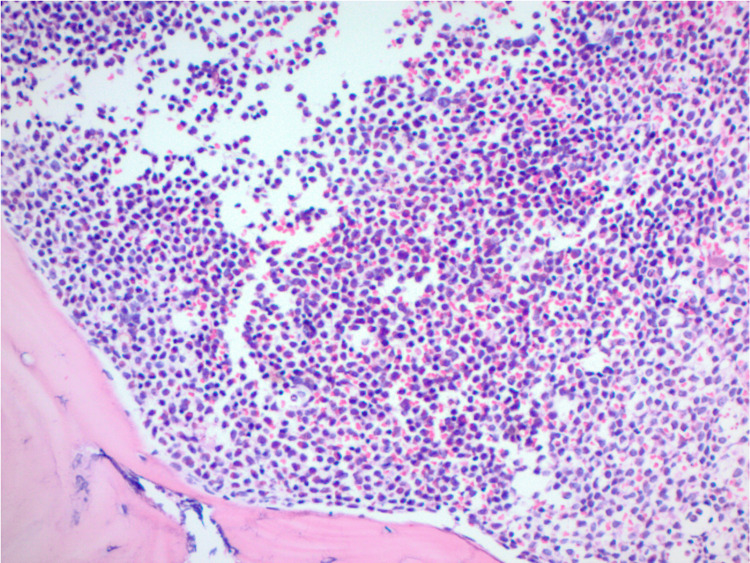
Morphology of bone marrow core biopsy Hypercellular marrow with diffuse proliferation of atypical promyelocytes with pink (eosinophilic/granular) cytoplasm at 20× magnification.

**Figure 3:**
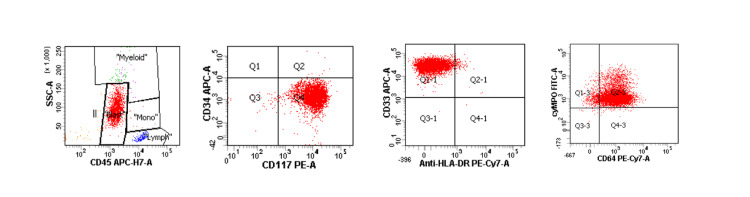
Representative flow cytometry dot plot of bone marrow aspirates Abnormal promyelocytes with high side scatter, positive for CD117, CD33, CD64, and cytoplasmic myeloperoxidase, and negative for CD34 and HLA-DR (APL context: CD33, CD117, CD64, and myeloperoxidase are typically positive, whereas CD34 and HLA-DR are typically negative). APL, acute promyelocytic leukemia; CD, cluster of differentiation; HLA-DR, human leukocyte antigen-DR isotype

The patient remained hospitalized for the next two weeks with a minimal hematologic response and continued pancytopenia. Given the patient’s history of gastric bypass, impaired absorption of ATRA was considered because a treatment response was anticipated by this point. Therefore, therapy was adjusted to sublingual ATRA 40 mg twice daily with continued intravenous arsenic trioxide. Given that there is no commercially available sublingual ATRA, the patient was instructed to administer the medication by piercing the capsule with her teeth and keeping the contents sublingually until absorption.

After switching to sublingual ATRA, the patient showed marked improvement within several days in regard to her complete blood count parameters, as demonstrated in Table 2. After concluding induction ATRA and arsenic trioxide, the repeat bone marrow biopsy was morphologically negative for residual APL, and she was ultimately discharged. The patient completed seven cycles of ATRA and four outpatient cycles of consolidation arsenic trioxide, with undetectable PML::RARA noted on repeat bone marrow biopsy. She continues to be in clinical remission two years after completing therapy.

**Table 2:** Complete blood count four days after switching to sublingual administration of ATRA, showing markedly improved counts across most cell lines ATRA, all-trans retinoic acid

	Normal limits	Actual count
Platelets	150-450 K/uL	150 k/uL (normal)
White blood cell count	3.8-10.6 K/uL	4.4 k/uL (normal)
Absolute neutrophil count	1.8-7.7 K/uL	2.2 k/uL (normal)
Hemoglobin	13.5-17.0 g/dL	8.1 g/dL (low)

Case 2

A 39-year-old man presented to the ED with right-sided paresthesia. He had a history of obesity and had undergone gastric bypass surgery about six months earlier. Magnetic resonance imaging of the brain revealed scattered subarachnoid hemorrhage along the right frontal convexity, as shown in Figure 4. Laboratory tests revealed low hematologic parameters, as demonstrated in Table 3. Peripheral smear showed 40% blasts and promyelocytes, raising concern for APL, which was confirmed through bone marrow biopsy analysis (80% blasts and abnormal promyelocytes on differential) and molecular testing.

**Figure 4:**
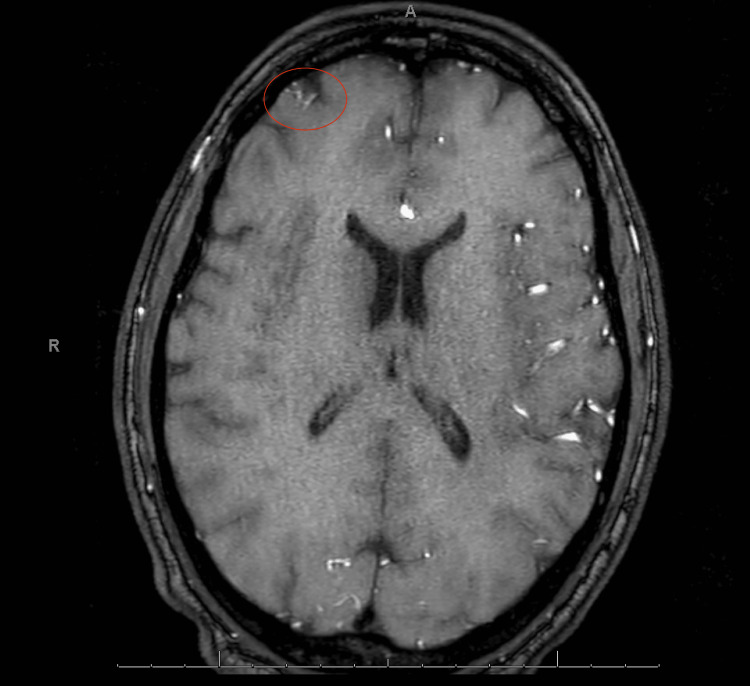
Brain MRI performed on admission because of neurologic deficits The red circle highlights a cross-section of subarachnoid hemorrhage along the right frontal convexity. MRI, magnetic resonance imaging

**Table 3:** Complete blood count Pancytopenia was present on admission across all cell lines. Differential notable for 3% blasts.

	Normal limits	Actual count
Platelets	150-450 K/uL	28 K/uL (low)
White blood cell count	3.8-10.6 K/uL	25.8 K/uL (high)
Blast %	0%	3% (high)
Hemoglobin	13.5-17.0 g/dL	6.4 g/dL (low)

The patient was started on oral ATRA (50 mg twice daily) and arsenic trioxide, and he received one dose of gemtuzumab ozogamicin 6 mg/m². However, the patient had a minimal response after approximately 10 days, with complete blood count showing persistent pancytopenia without white cell differentiation.

Given the patient’s history of gastric bypass, inadequate ATRA absorption was suspected. Therefore, self-administered sublingual ATRA was started. The patient showed modest hematologic improvement. Within four days after switching to sublingual ATRA, his blood counts showed marked improvement, as seen in Table 4.

**Table 4:** Complete blood count four days after transition to sublingual ATRA, showing improved hematologic parameters ATRA, all-trans retinoic acid

	Normal limits	Actual count
Platelets	150-450 K/uL	52 K/uL (low)
White blood cell count	3.8-10.6 K/uL	4.6 K/uL (normal)
Blast %	0%	3% (high)
Hemoglobin	13.5-17.0 g/dL	9.0 g/dL (low)

The patient received consolidation therapy with ATRA and arsenic trioxide in the outpatient setting. After five cycles, a repeat bone marrow biopsy confirmed morphologic, cytogenetic, and molecular remission after seven months, and the patient continues to be in remission two years after treatment completion.

## Discussion

Amazing strides in APL therapies have been made, starting with the introduction of ATRA in the 1980s and arsenic trioxide in the 1990s, which have led to improved remission rates exceeding 90% and cure rates greater than 80% [4]. Oral ATRA is approximately 50% bioavailable and acts by promoting autophagy and restoring normal hematopoietic differentiation; however, suboptimal systemic absorption of oral ATRA in some patients may diminish this drug’s effectiveness. Sublingual administration in these cases could theoretically prove more effective by bypassing first-pass gut absorption.

Several instances of sublingual and alternative ATRA administration have been reported, including in a pediatric patient who developed acute appendicitis and paralytic ileus during induction therapy for APL. With sublingual ATRA, the patient achieved clinical remission and remained relapse-free for three years [5]. However, the first documented case of sublingual ATRA administration occurred in 1996 in a comatose patient, establishing the strategy of administering ATRA sublingually [5,6]. Additionally, two patients with APL who were unable to swallow were given ATRA as an oil suspension via nasogastric tube, alongside daunorubicin, with favorable results [7].

For our patients, given their delayed clinical responses and histories of gastric bypass, sublingual ATRA was administered because of concerns regarding malabsorption. Notably, plasma ATRA levels are not typically measured during APL treatment, and whether sublingual ATRA administration improves systemic drug availability remains hypothetical [6]. Regardless, considering our patients’ rapid remission and other compelling evidence in the literature, clinicians should maintain awareness of sublingual ATRA administration for patients who have undergone gastric procedures that could affect oral ATRA effectiveness. Studies to define the systemic bioavailability of ATRA through a sublingual route are needed to optimize APL treatment protocols for patients who may be at risk for drug malabsorption.

## Conclusions

ATRA is a highly effective therapy for APL; however, ensuring adequate drug absorption in patients with altered gastrointestinal anatomy may require sublingual administration. Here, we describe two patients with previous gastric bypass who developed APL and were treated successfully with sublingual ATRA. Further studies are needed to validate the pharmacokinetics and efficacy of sublingual ATRA administration as an effective alternative route in patients with APL.
